# A patient‐ and acquisition‐tailored injection approach for improving consistency of CT enhancement towards a target CT value in coronary CT angiography

**DOI:** 10.1002/acm2.13867

**Published:** 2022-12-20

**Authors:** Gert Van Gompel, Laurence Delombaerde, Federica Zanca, Kaoru Tanaka, Dries Belsack, Johan de Mey, Nico Buls

**Affiliations:** ^1^ Vrije Universiteit Brussel (VUB), Universitair Ziekenhuis Brussel (UZ Brussel), Department of Radiology Brussels Belgium; ^2^ KU Leuven, Department of Oncology Leuven Belgium; ^3^ Palindromo Consulting Leuven Belgium

**Keywords:** body size metric, coronary CT angiography, contrast media, fat free mass, patient‐tailored contrast injection

## Abstract

**Background:**

Unoptimized coronary CT angiography (CTA) exams typically result in a highly variable arterial enhancement (HU_a_) across patients. This study aimed at harmonizing arterial enhancement by implementing a patient‐, contrast‐ and kV‐tailored injection protocol.

**Methods:**

First, the optimal body size metric to predict HU_a_ was identified by retrospectively analysing images of 76 patients, acquired with 70 ml contrast media (G1). Second, using phantom experiments, correction factors for the effect of kV and contrast concentration on HU_a_ were determined. Third, a model was developed, prescribing the optimal contrast dose to be injected to obtain a diagnostically appropriate arterial target enhancement HU_target_. The model was then validated on 278 prospectively collected patients, in two groups with two different HU_target_: 525 HU (207 patients, G2A) and 425 HU (71 patients, G2B). The HU_a_ histograms were compared among groups and to the target enhancement through their mean and standard deviation (SD) at 100 kVp reference level. Also, signal‐to‐noise ratio was obtained and compared among the groups.

**Results:**

Fat free mass (FFM) showed the highest correlation with HU_a_ (*r* = 0.69). KVp correction factors ranged from 0.65 at 70 kVp to 1.22 at 140 kVp. The obtained model reduced the group heterogeneity (SD) from 101HU for reference G1 to 75HU (*p* < 0.001) for G2A and 68HU (*p* < 0.001) for G2B. The mean HU_a_ of 506HU in G2A was slightly below HU_target_ = 525HU (*p* = 0.01) whereas in G2B, the mean HU_a_ of 414HU was not significantly different from HU_target_ = 425HU (*p* = 0.54). The total iodine dose was lowered from 19.5 g‐I to 17.6 g‐I and 14.2 g‐I from G1 to G2A and G2B, on average.

**Conclusion:**

A contrast injection model, based on patient's fat free mass and accounting for the contrast agent concentration and the planned CT‐scan tube voltage, harmonized arterial enhancement among patients towards a predefined target enhancement in coronary CTA scanning, without affecting the bolus timing.

## 1 INTRODUCTION

Coronary CT angiography (CTA) has become the most important tool for the detection of coronary artery disease for patients presenting with stable chest pain. Recent evolutions in CT technology, such as single‐heartbeat scanning, iterative reconstruction and automatic kVp selection, dramatically improved coronary CTA acquisition in terms of image quality, radiation dose and acquisition duration.

Injection protocols on the other hand, which also have a critical impact on image enhancement, have not yet fully followed this evolution. CTA image enhancement by HU varies with body habitus and kVp selection. Ideally, each patient would be given the necessary amount of contrast media to obtain an appropriate enhancement.^1^, ^2^ A range between 325 and 500HU is considered optimal to avoid low attenuation scans (<325HU) as well as underestimation of stenosis and plaque cross‐sectional area due to blooming artifacts associated with high enhancement (>500HU).^2^, ^3^ It is considered good clinical practice to individualize the necessary CM dose for each patient as CM administration is associated with potential adverse effects such contrast induced nephropathy (CIN),^4^, ^5^ of which the prevalence appears to be CM dose dependent.^6^, ^7^ In addition to these adverse effects, recent publications have shown an increased radiation dose in blood, and potentially also organs^8^ related to CM dosing.^9^

To harmonize intra‐arterial enhancement among patients towards a target level, the injection protocol optimization should account for all parameters affecting image enhancement, that is, the CT scanning factors (kVp, scan timing), contrast administration factors (iodine concentration, injection rate and volume) and patient related factors such as body habitus and cardiac output. Several studies have proposed compensation factors to adapt the iodine volume to the used kVp, either to preserve intra‐arterial enhancement^10^, ^11^ or CNR/SNR.^11^, ^12^

Regarding body habitus tailored contrast injection, it is still a subject of investigation which body metric, or combination of body metrics, are key in predicting the degree of image enhancement. Some studies mention a significant correlation between BMI and image enhancement.^1^ A higher impact on interpatient CT value consistency in coronary CTA was reported for height, and especially for weight and body surface area (BSA).^1^, ^13^, ^14^, ^15^, ^16^ These studies were typically focused on a single aspect of the optimization and/or were limited to either a retrospective analysis or a prospective study based on an empirical non‐optimized model. In this study, we propose a generalized model for patient‐tailored contrast injection to reduce interpatient variability of arterial enhancement and to target a predefined image enhancement during single‐heartbeat coronary CTA, accounting for kVp, CM concentration, patient body size, without impact on bolus‐ or scan timing. The model was based on a retrospective analysis and tested in a prospective study.

## 2 METHODS

### 2.1 Patient collective

**FIGURE 1:**
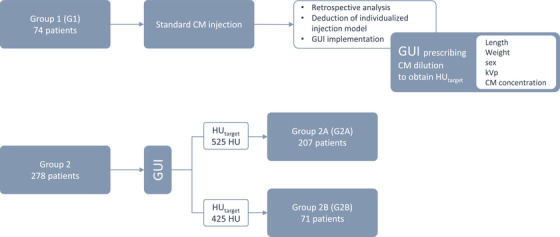
Patient enrolment and study outline

The study was approved by the local medical ethics committee (BUN 143201524614). A total of 352 patients, suspected of coronary artery disease and referred to the radiology department for a coronary CTA, were enrolled (Figure 1). Seventy‐four cases (group 1, G1) with coronary CTA scans were retrospectively analysed to establish a generalized contrast injection model. The model was tested in a prospective study, subdivided in two groups with different target arterial CT numbers (HU_target_): 207 patients (group 2A, G2A) with HU_target_ = 525 HU, and 71 patients (group 2B, G2B) with HU_target_ = 425 HU. Demographical patient data (sex, age, height and weight), details from the scan protocol (radiation dose as dose‐length product (DLP) and computed CT dose index (CTDI_vol_), and tube potential) and iodine contrast agent information (volume, concentration, vendor) were collected.

### 2.2 CT acquisition and evaluation

All patients (G1, G2A and G2B) were scanned with the local standard‐of‐care coronary CTA scan protocol on a 256‐slice CT system (Revolution CT, GE Healthcare). The protocol consists of a single‐rotation axial scan with prospective gating, 16 cm detector coverage, noise index 30 (keeping the noise level fixed over all kVp levels and body sizes), and gantry rotation time 0.28 s. Tube potential ranged between 80 and 100 kV depending on body size. A 7s scan delay was set after automatic bolus tracking, triggered at an increased iodine enhancement of 100 HU in the descending aorta. Heart rate was controlled by oral beta‐blockers (Seloken 5 mg, AstraZeneca).

Arterial enhancement was evaluated on a clinical workstation (IMPAX, Agfa Healthcare), in the axial slices reconstructed at 75% of the cardiac cycle (R‐R). The average arterial CT number HU_a_, and standard deviation (SD) were measured, and signal‐to‐noise ratio was obtained in a circular ROI in the ascending aorta at the level of the left coronary artery branch.

### 2.3 Contrast model setup

#### 2.3.1 Influence of patient body size (G1)

The following body metrics were considered to study the impact on arterial enhancement: body mass index (BMI (kg/m^2^), height (H (m)), body weight (BW (kg)), body surface area (BSA (m^2^)),^17^ lean body mass (LBM (kg))^18^ and fat‐free mass (FFM (kg)), reflecting the total body weight without adipose tissue^19^ (Table 1).

**TABLE 1:** Definitions of BSA, LBW and FFM

Descriptor	Formula	
Body surface area	$BSA\left[m^{2}\right]=\sqrt{BW\cdot H\left[\mathrm{cm}\right]}/60$	
Lean body weight	*LBW*[kg] = 0.32810 · *BW*[kg] + 33.929 · *H*[m] – 29.5336	for men
	*LBW*[kg] = 0.29569 · *BW*[kg] + 41.813 · *H*[m] – 43.2933	for women
Fat free mass	$\mathrm{FFM}\left[\mathrm{kg}\right]=0.285\cdot \mathrm{BW}\left[\mathrm{kg}\right]+12.1\cdot H{\left[m\right]}^{2}$	for men
	$\mathrm{FFM}\left[\mathrm{kg}\right]=0.287\cdot \mathrm{BW}\left[\mathrm{kg}\right]+9.74\cdot H{\left[m\right]}^{2}$	for women

In the retrospective study, the administered total iodine doses (TID) were normalized with the patient‐specific body metrics (TID/BSA, TID/BMI, TID/BW, TID/height, TID/LBM, TID/FFM) to investigate their correlation with the average arterial CT number.

#### 2.3.2 Influence of tube potential and (kVp) and iodine concentration: phantom study

To investigate the effect of kVp and contrast concentration on image enhancement, a contrast agent iodixanol 320 mgI/ml (Visipaque, GE Healthcare) was diluted using a standard saline solution (0.9% NaCl, Viaflo, Baxter). Four rods were filled with iodine solutions with concentrations 2, 4, 8 and 16 mg I/ml and inserted in a cylindrical water tank (AAPM CT Performance Phantom Model 610, CIRS). Images were acquired at 70, 80, 100, 120 and 140 kVp. The relation of kVp and iodine concentration with CT enhancement (by HU) was investigated and correction factors C_kVp_ were derived that compensate for changes in kVp.

#### 2.3.3 Model for optimal and personalized iodine dose injection

By combining this correction factor C_kVp_ with the body size metric most highly correlated with the target arterial enhancement, a model was developed that allows the calculation of the iodine dose required to obtain a predefined diagnostically acceptable target value (HU_target_) of arterial CT enhancement.

### 2.4 Contrast injection

#### 2.4.1 Retrospective study

For all patients in G1, CM was injected into the antecubital vein following the local standard‐of‐care tri‐phasic injection protocol, with fixed injection rate of 5 ml/s. In the first phase, a fixed amount (55 ml) of CM is administered, followed by 38 ml of a diluted solution (40% CM – 60% saline) (phase 2) for artifact‐free visualization of the right ventricular cavity,^20^ and subsequently 30 ml saline chaser (phase 3).

Four different contrast media were used in a weekly rotation: Iodixanol 320 mg I/ml (Visipaque, GE Healthcare, Cork, Ireland), Iomeprol 350 mg I/ml (Iomeron, Bracco, Milan, Italy), Iobitridol 350 mg I/ml (Xenetix, Guerbet, Paris, France) and 370 mg I/ml (Ultravist, Bayer, Leverkusen, Germany).

#### 2.4.2 Prospective study

In the prospective study, phase 1 of the injection protocol was altered by diluting the CM with saline so as to inject the optimal iodine concentration (mgI/ml) to result in a predefined target arterial enhancement (HU_target_). Two target enhancements were investigated: a conservative HU_target_ value of 525 HU (G2A), as well as a value of 425 HU (G2B) which falls within the ideal range for enhancement of the coronary arteries from 325 to 500 HU.^2^, ^3^, ^21^ The total injected volume was kept fixed at 55 ml delivered at a rate of 5 ml/s to avoid an impact on the time‐to‐peak of the time‐enhancement curve. As an exception, high FFM‐patients with estimated optimal dilution percentages exceeding 100% received prolonged injection of undiluted CM until the optimal total iodine dose (TID) was delivered.

### 2.5 Graphical user interface and injection workflow

The contrast injection model was implemented in a standalone graphical user interface (GUI) using HyperNext Studio (freeware) and installed at a PC in the CT console room to simplify the workflow. After entering the kV, patient FFM, and contrast concentration parameters into the GUI, the required CM dilution percentage was displayed and could be directly entered at the CT console, which controlled a Class IV dual head power injector (Dual shot GX7, Nemoto‐Kyorindo).

### 2.6 Statistical analysis

All statistical analysis was performed with SPSS 20.0 (IBM SPSS Statistics). Linear regression with Pearson product moment correlation coefficient (r), was performed to determine the strength of association between CT attenuation in the aorta and the considered body size descriptors. The regression was constrained at the non‐enhanced CT value of blood (50 HU). Correlation strength is categorized following Evans: 0.00–0.19 very weak, 0.20–0.39 weak, 0.40–0.59 moderate, 0.60–0.79 strong, 0.80–1.0 very strong. Statistical differences between correlations from dependent samples were evaluated following Steiger,^22^, ^23^ and were considered significant with *p*‐values ≤ 0.05.

Normality of and differences in patient demographics were tested using the Shapiro Wilk and Kruskal‐Wallis test. Mann‐Whitney U testing was applied to compare enhancement (HU) and total iodine dose between groups, with stratified analysis per gender. Levene's test was used to compare homogeneity of variances.

## 3 RESULTS

### 3.1 Patient collective

There were no significant differences in demographic, patient, and acquisition characteristics of G1, G2A and G2B in terms of sex, age, BW, BMI, BSA, FFM, heart rate and radiation dose (Table 2)

**TABLE 2:** Demographic, patient and acquisition characteristics

Characteristic	G1 standard	G2A HU_target_ = 525HU	G2B HU_target_ = 425HU	*p*‐value
Number of patients	74	207	71	
Male/female	39/35	85/122	32/39	
Age (years)	52.3 ± 11.6	55.8 ± 13.3	54.7 ± 12.9	*p* = 0.15
Height (cm)	168.8 ± 9.7	168,66	170,9 ± 11.3	*p* = 0.17
Weight (kg)	76.5 ± 15.5	75.0 ± 15.9	77.5 ± 17.2	*p* = 0.39
BMI	26 ± 5	26 ± 5	27 ± 5	*p* = 0.76
BSA (m^2^)	1.89 ± 0.22	1.86 ± 0.22	1.91 ± 0.25	*p* = 0.30
FFM (kg)	53.9 ± 9.7	52.1 ± 8.9	54.2 ± 10.8	*p* = 0.27
Heart Rate (bpm)	62.3 ± 12.8	60.4 ± 9.9	59.1 ± 10.1	*p* = 0.35
Dose‐Length Product (mGy*cm)	120.7 ± 78	118.2 ± 68	103.9 ± 63	*p* = 0.08
CTDI_vol_ (mGy)	20.4 ± 7.2	20.5 ± 9.0	17.0 ± 4.7	*p* < 0.001

### 3.2 Influence of tube potential (kVp) and iodine concentration on enhancement

**FIGURE 2:**
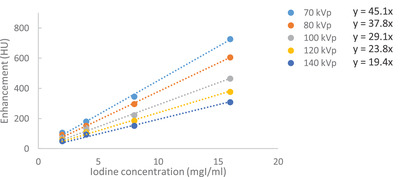
Regression of CT value (HU) versus iodine concentration for 70–140 kVp

A linear relation was observed between enhancement and iodine concentration in the clinical range for each of the kVp's (Figure 2). The corresponding kVp correction factors C_kVp_ for the iodine concentration to preserve enhancement (HU) with reference to 100 kVp ranged from 0.65 (70 kVp), 0.77 (80 kVp), 1.00 (100 kVp), 1.22 (120 kVp) to 1.50 (140 kVp).

### 3.3 Image quality

#### 3.3.1 Retrospective study: Influence of sex and body size on enhancement

**FIGURE 3:**
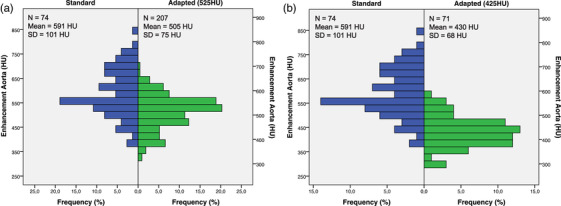
Intra‐arterial enhancement distribution in the retrospective study (G1, in blue) compared to enhancement for (a) G2A with HU_target_ = 525 HU (green), and for (b) G2B with HU_target_ = 425 HU (green). The blue and green shaded bars indicate the 2 SD range of each distribution

Analysis of enhancement in G1 (Figure 3, blue distribution) showed a normal distribution of the arterial enhancement, with mean value 590 HU and SD 101 HU. An inverse and significant (*p* < 0.001) correlation was found between arterial enhancement and body metric normalized total iodine dose for all body metrics. We observed a weak correlation (*r* = 0.31) for BMI, a moderate correlation for height (*r* = 0.47), and strong correlations for BW (*r* = 0.62), BSA (*r* = 0.63), LBM (*r* = 0.68) and FFM (*r* = 0.69). FFM and LBM showed the highest correlation, both significantly higher (*p* ≤ 0.05) than BW, BSA, height and BMI.

**FIGURE 4:**
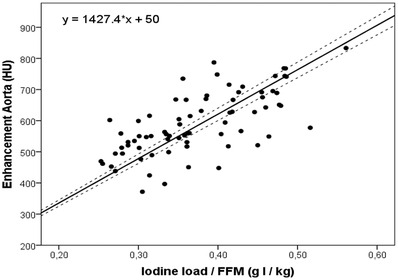
Constrained regression (solid) with the 95% confidence interval (dashed) of intra‐arterial attenuation (HU) versus the FFM‐normalized total iodine dose

Adopting the corresponding linear regression with FFM (Figure 4), the required total iodine dose TID (mg‐I) to reach the target enhancement HU_target_ at 100 kVp is written as:

(1) $$\mathrm{TID}\;=\mathrm{FFM}\;\frac{HU_{\mathrm{target}}-50}{1.43}$$

Given the CM iodine concentration CM_conc_(mgI/ml), kVp compensation factor C_kVp_, and a fixed total injection volume V_inj_ = 55 ml, the dilution percentage of CM (%_CM_) with saline becomes:

(2) $${\%}_{CM}=100\frac{\mathrm{TID}\;}{CM_{Conc}.V_{\mathrm{inj}}}\;C_{kVp}$$

#### 3.3.2 Prospective study

**FIGURE 5:**
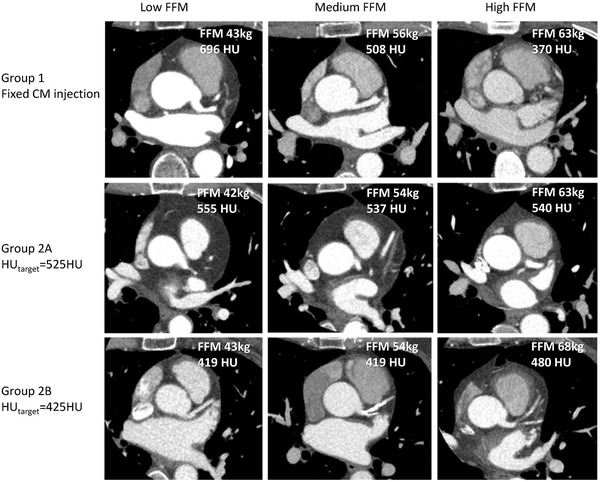
Illustrative coronary CTA images from G2A and 2B (tailored protocol) versus the reference G1 with fixed CM injection, for patients with similar BMI, in three FFM categories: low (left column), medium (centre column), and high FFM (right column)

Figure 5 illustrates coronary CTA images from the tailored protocol (G2A and G2B) versus the standard CM injection (G1), for patients with similar BMI and low, medium and high FFM. Table 3 summarizes the used CM volume, total iodine and the resulting mean CT number HU_a_ and SNR in aorta for G2A and G2B in comparison with G1. HU_a_ was normally distributed (*p* = 0.02 and *p* = 0.59, respectively) in G2A and G2B (Figure 3, green distributions). In G2A and G2B, HU_a_ was 505 HU (lower than the HU_target_ = 525 HU (*p* < 0.01)) and 430 HU (not significantly different from HU_target_ = 425 HU, *p* = 0.54), with, respectively, 42.2% and 80.3% of the cases reaching HU_a_ values within the optimal range (325 HU—500 HU), compared to 18.9% for G1. Low image quality in terms of low HU_a_ (<325 HU) was found in 0.0%, 0.5% and 5,6% of the cases for G1, G2A and G2B, respectively.

**TABLE 3:** Injected CM volumes, total iodine dose, and CT number and SNR in ascending aorta, for the three patient groups

	Injection approach	sex	# cases	CM volume	total iodine dose	Mean CT number aorta HU_a_ (± stdev)	Mean SNR aorta (± stdev)
G1	standard	M	39	55,0	19,4 (± 1,3)	511,7 (± 76,1)	14.4 (± 2.3)
		F	35	55,0	19,6 (± 1,3)	620,1 (± 90,7)	14.5 (± 2.3)
		M+F	74	55,0	19,5 (± 1,3)	567,3 (± 100,9)	14.4 (± 2.3)
G2A	model (GUI) HU_target_ = 525	M	84	58,9 (± 9,1)	20,7 (± 3,0)	523.6 (± 74,9)	13.7 (± 2.3)
		F	123	43,9 (± 7,1)	15,5 (± 2,4)	493,4 (± 71,7)	13.1 (± 2.2)
		M+F	207	50 (± 10,9)	17,6 (± 3,7)	505,8 (± 74,3)	13.4 (± 2.2)
G2B	model (GUI) HU_target_ = 425 HU	M	32	47,8 (± 4,9)	16,8 (± 1,6)	417,3 (± 72,1)	12.3 (± 1.8)
		F	39	34,2 (± 3,8)	12,0 (± 1,3)	398,4 (± 64,7)	11.5 (± 1.7)
		M+F	71	40,3 (± 8,0)	14,2 (± 2,8)	413,9 (± 68,7)	11.9 (± 1.8)

As the acquisition protocol and noise level was kept constant using a fixed noise index, SNR decreased corresponding with the mean attenuation from 14.4 for G1 to 13.4 and 11.9 for G2A and G2B.

Compared to the heterogeneity of enhancement (SD = 101HU) in G1, the standard deviations have been reduced significantly for G2A (75HU (*p* < 0.001)) and G2B (68 HU) (*p* < 0.001). The maximum enhancement was decreased from 833HU (G1) to 717(HU) (G2A) and 589HU (G2B). SNR homogeneity in terms of standard deviation among the groups improved from 2.3 (G1) to 2.2 (G2A) and 1.8 (G2B).

**FIGURE 6:**
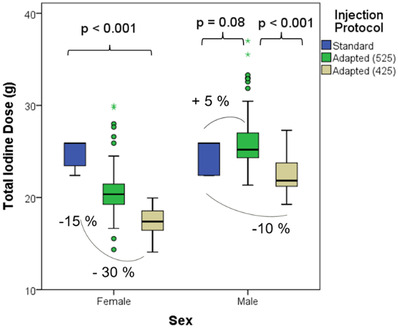
Subset analysis of total iodine dose. Boxplot of TID (g‐I) used in G1 (blue), G2A with HU_target_ = 525 HU (green) and G2B with HU_target_ = 425 HU (yellow)

The total iodine dose of the first and second injection phase was significantly lower (*p* < 0.001) for women in G2A (15.5 g) and G2B (12.0 g) compared to the standard protocol of G1 (19.3 g) (Figure 6). For men, a lower (*p* < 0.001) average group iodine dose was obtained for G2B (16.8 g), but not for G2A (*p* = 0.08) (20.7 g) compared to G1 (19.6 g).

## 4 DISCUSSION

This study aimed at developing and validating a model for contrast injection that reduces intra‐arterial enhancement heterogeneity among patients, by accounting for the CT scan factors, CM agent concentration and the patient's fat‐free mass. The model is set up to be tunable in an intuitive way and to target a predefined enhancement.

Several authors have reported results on low kVp scanning to reduce radiation and iodine dose in contrast enhanced CT.^10^, ^11^, ^24^, ^25^ The VOLCANIC study 12 showed that by applying kVp correction factors in an auto‐kVp imaging protocol, the CNR and subjective image quality in the patient group can be adequately preserved. The magnitude of the kVp correction factors found in our study (from 0.65 at 70 kVp to 1.22 at 140 kVp) indicates the importance of adapting the CM injection to the planned kVp of the coronary CTA scan. The obtained factors are in close agreement with Bae et al.,^10^ but differ somewhat from Higashigaito,^11^ due to the different beam prefiltration on CT systems.

Regarding body size correction, we found retrospectively that arterial enhancement HU_a_ correlates significantly with iodine dose if it is weighted with (in increasing order) BMI, height, BW, BSA, LBM, and FFM. Earlier studies also reported some degree of CT enhancement levelling among patients in case of BMI‐ ^1^, ^26^ and BW‐ ^10^, ^15^, ^16^ scaled iodine administration. The higher correlation of BSA and LBM‐weighted volume with HU_a_ confirms the results by Bae et al.^1^, Yanaha et al.^27^ and Eijsvoogel et al.^21^, and can be explained by a smaller dependence on body fat and an improved correlation with the cardiac output, which is known to relate to image enhancement.

In addition, we found that the injected iodine volume is significantly better correlated to image enhancement if it is adapted to the FFM or LBM, both accounting for the patient sex, compared to BSA. These observations are supported by Green et al.,^28^ stating that FFM and LBM improve medicine dosing compared to BSA, by Collis et al.^29^ who found that FFM is more related to cardiac output than BSA, and by Lembcke et al.^30^ who observed a positive impact of a sex‐specific CM injection approach on the constancy of enhancement among patients.

With a GUI‐based clinical workflow, the individualized injection approach was easily accepted in our radiology department as the new standard coronary CTA protocol, with HU_target_ appreciated as an intuitive tuning parameter. The prospective study showed with a mean group enhancement of 505 HU for HU_target_ = 525HU, and 430 HU for HU_target_ = 425 HU, that this parameter effectively indicates the average outcome enhancement of a patient group. The group with target value 425 HU yielded the largest portion (80%) of image enhancements within the optimal range (325–500 HU), but nevertheless a slight increase of this target value might be desirable to eliminate the majority of the 5.6% of low attenuation cases (<325 HU).

The TID was lowered from 25 g‐I with the standard protocol to 20 g‐I with HU_target_ = 425 HU, which is close to the results of Nakaura et al.^16^ (22g for BW‐based iodine administration), despite the much larger average body weight of the population in our study (74 kg vs. 55 kg). The TID was different (*p* < 0.001) between sexes: the same HU_target_ = 425 HU required a mean TID of 18 g‐I for women compared to 22 g‐I for men.

Our study used a bolus tracking technique. Alternatively, with a pre‐bolus approach that allows to synchronize the scan timing with the arterial peak, it is reported that even lower interpatient variability can be achieved down to 11% and 7% for a European and an Asian population, respectively,^2^ compared to 14%–16% in this study. However, despite the potential of improved scan timing of such approach in comparison with bolus tracking, its implementation in a clinical context is considered to be more cumbersome^2^ and requires an additional contrast bolus of around 20%. A dual‐ROI tracking approach, with an ROI in ascending aorta and the pulmonary trunc^31^ could combine the advantages of timing bolus scanning with bolus tracking.

The following limitations should be considered. Firstly, this study evaluates enhancement and enhancement consistency, but it does neither recommend a certain target enhancement value, nor evaluate image quality in terms of CNR or expert reading analysis. When implementing the proposed model in a clinical CCTA imaging protocol, these additional image quality metrics should be carefully evaluated. Secondly, the model was set up based on our local tri‐phasic injection protocol for right heart visualization^32^ of which only the first phase is adjusted to patient, kVp and CM concentration. Although Wuest et al.^33^ found that the 2nd phase has very limited impact on coronary enhancement, additional validation would be required when omitting or patient‐tailoring the 2nd phase.

Thirdly, the proposed approach is generalized with respect to kVp, contrast concentration and patient habitus, targeting a peak enhancement without affecting the peak timing. The regression values, however, are specific to the injection protocol (rate, volume), scan timing and CT filtration. Finally, we used measurements in ascending aorta as a surrogate for the coronary arteries, because of improved reliability of average HU values associated with the larger ROI size. Implementation of this method in a clinical setting requires careful evaluation of the coronary enhancement.

## 5 CONCLUSION

In conclusion, we found in a retrospective comparative study that fat‐free mass, among body weight, height, BMI, BSA and LBM, is the optimal body size descriptor to scale iodine volume administered to the patient. We set up and validated a model for contrast injection in coronary CTA accounting for the planned CT scan tube potential, contrast media concentration and the body habitus reflected by the FFM, and drastically reduced the interpatient intra‐arterial enhancement variability versus a fixed injection protocol. To our knowledge, it is the first time that a generalized iodine‐delivery‐rate optimization approach, which is tunable towards a target CT enhancement, is proposed in combination with bolus tracking. The model‐GUI can be obtained by contacting the authors.

## AUTHOR CONTRIBUTIONS

The study was conceptualized and the methodology was set up by NB, FZ, GVG and LD. GVG and LD analyzed the data; all authors were involved in the data interpretation. GVG and LD were the major contributors to the manuscript, which was thoroughly reviewed by NB, JdM, KT, DB and FZ. All authors read and approved the final manuscript.

## CONFLICT OF INTEREST

No conflict of interest.

## ETHICS STATEMENT

The study was approved by the medical ethics committee UZ Brussel (BUN 143201524614).

## Data Availability

The datasets generated during the current study are not publicly available to protect the patients privacy, but are available from the corresponding author on reasonable request.
